# Statistical analysis of timeseries data reveals changes in 3D segmental coordination of balance in response to prosthetic ankle power on ramps

**DOI:** 10.1038/s41598-018-37581-9

**Published:** 2019-02-04

**Authors:** Nathaniel T. Pickle, Anne K. Silverman, Jason M. Wilken, Nicholas P. Fey

**Affiliations:** 1 Department of Bioengineering, The University of Texas at Dallas, Richardson, TX 75080 USA; 2 Department of Mechanical Engineering, Colorado School of Mines, Golden, CO 80401 USA; 3 Center for the Intrepid, Brooke Army Medical Center, JBSA Ft Sam Houston, TX 78234 USA; 4 Extremity Trauma and Amputation Center of Excellence, JBSA Ft Sam Houston, TX 78234 USA; 5 Department of Physical Medicine and Rehabilitation, The University of Texas Southwestern Medical Center, Dallas, TX 75390 USA

## Abstract

Active ankle-foot prostheses generate mechanical power during the push-off phase of gait, which can offer advantages over passive prostheses. However, these benefits manifest primarily in joint kinetics (e.g., joint work) and energetics (e.g., metabolic cost) rather than balance (whole-body angular momentum, *H*), and are typically constrained to push-off. The purpose of this study was to analyze differences between active and passive prostheses and non-amputees in coordination of balance throughout gait on ramps. We used Statistical Parametric Mapping (SPM) to analyze time-series contributions of body segments (arms, legs, trunk) to three-dimensional *H* on uphill, downhill, and level grades. The trunk and prosthetic-side leg contributions to *H* at toe-off when using the active prosthesis were more similar to non-amputees compared to using a passive prosthesis. However, using either a passive or active prosthesis was different compared to non-amputees in trunk contributions to sagittal-plane *H* during mid-stance and transverse-plane *H* at toe-off. The intact side of the body was unaffected by prosthesis type. In contrast to clinical balance assessments (e.g., single-leg standing, functional reach), our analysis identifies significant changes in the mechanics of segmental coordination of balance during specific portions of the gait cycle, providing valuable biofeedback for targeted gait retraining.

## Introduction

Advanced robotic leg prostheses for people with below-knee (transtibial) amputations have been developed^1^ that are capable of generating the same magnitude of mechanical power as a biological ankle during the push-off phase of gait^2,3^. This capacity to actively generate mechanical power during stance (when the foot is in contact with the ground) contrasts with conventional passive energy-storage-and-return prostheses, which absorb elastic energy during loading that is released passively during unloading. Although mechanically passive prostheses also generate positive power during push-off as they return stored elastic energy, their mechanical efficiency is typically only between 49 and 59%, depending on the stiffness of the foot and on the method of estimation^4^.

Benefits of mechanically active prostheses have been demonstrated in joint kinetics and metabolic cost. For example, studies have found that in comparison to passive prostheses, active prostheses produce increased peak ankle power^3,5^ and can reduce metabolic energy expenditure on level-ground^5,6^. However, other studies have found that mechanically active ankle-foot prostheses do not necessarily reduce metabolic cost, despite increased ankle push-off work^7^. In addition, the influence of active prostheses on joint kinetics appears to remain localized within the specific portion of the gait cycle (heel strike to consecutive heel strike of the same leg) when the device actively generates mechanical power^2^.

Furthermore, the observed improvements in energetic measures may come at the expense of balance. Dynamic balance (i.e., avoiding a fall during dynamic tasks such as walking) is commonly quantified using the peak-to-peak range of whole-body angular momentum (*H*) due to its correlation with balance impairment, as evidenced by frontal-plane *H* in elderly individuals with vestibular dysfunction^8^ and individuals post-stroke^9^. The range of *H* provides a single metric that captures whole-body coordination of balance and indicates the overall magnitude of net external moment required to regulate dynamic balance during a stride through the relationship

1 $$\dot{H}={\sum }^{}{M}_{ext}.$$

The ability to maintain dynamic balance is a key concern for people with transtibial amputation (TTA), as fear of falling is prevalent in this population^10^. The range of sagittal-plane *H* during prosthetic leg stance is greater in people with TTA using both passive and active prostheses compared to non-amputees at various walking speeds^11,12^ as well as on stairs^13^ and ramps^14^, suggesting either an impaired ability to regulate *H* or a willingness to compromise stability for the sake of other objectives.

Thus, while important advantages of active prostheses have been identified, it appears that there are limited improvements in balance and the kinetic benefits are temporally localized to push-off. Yet despite these biomechanical limitations of active prostheses, the issue is further complicated by studies finding that users prefer an active prosthesis over a passive one^3^. The discrepancy between quantitative biomechanical measures and qualitative user feedback suggests that it is important to consider not only local joint mechanics (e.g., prosthetic ankle power generation) or lumped whole-body measures (e.g., net metabolic energy expenditure, step-to-step transition work^5^, range of *H*) but also the effects of the device on segmental coordination during gait^15–17^. Here, we define “coordination” as the contributions of individual segments to whole-body movement at specific times during a movement.

**Figure 1:**
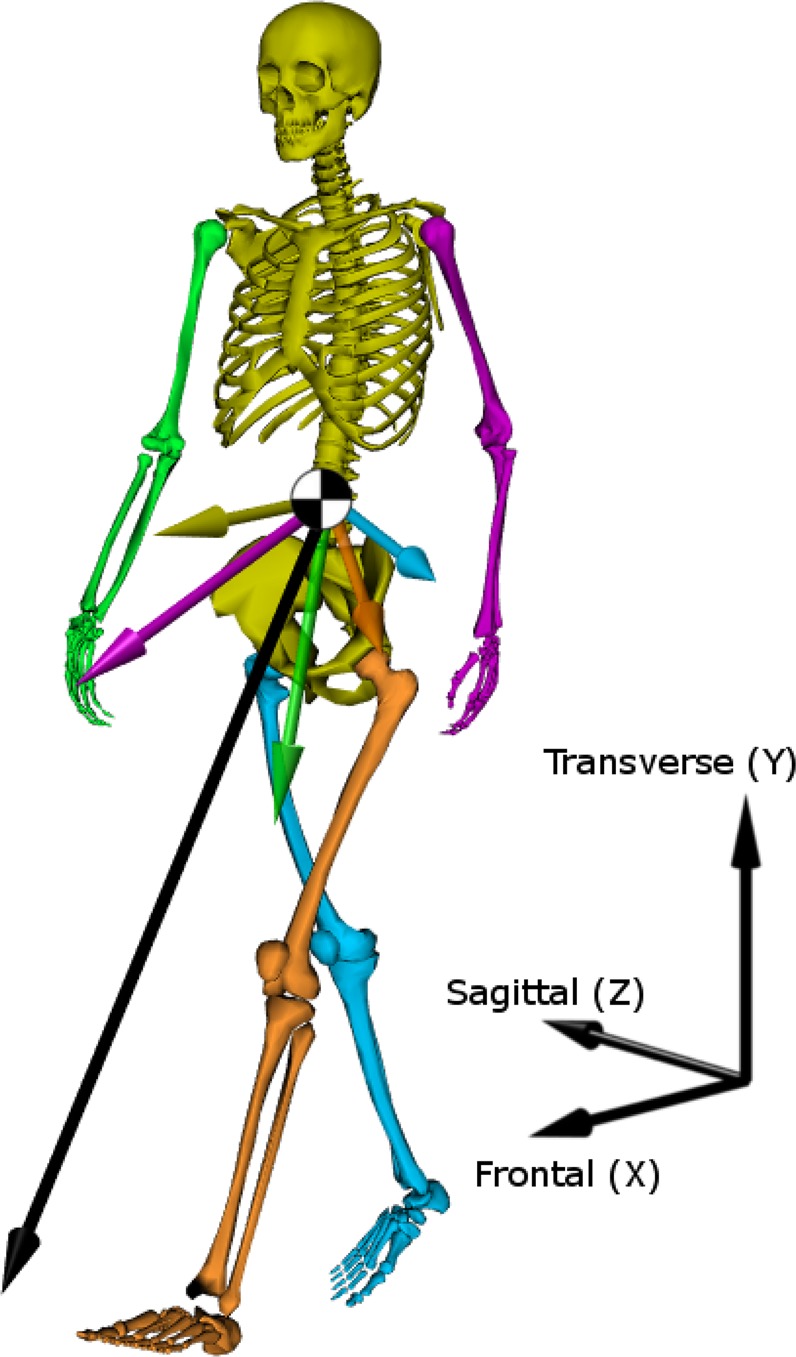
Illustration of the contributions of each body segment to total three-dimensional whole-body angular momentum (*H*) during walking. The three-dimensional contribution of each segment relative to the body center of mass is shown in the color corresponding to that segment. The total *H* is the sum of all segment contributions, and is shown with a black arrow originating at the center of mass. The size of the vectors has been scaled for clarity – typically the contributions of the arms are much smaller than the other segments. Skeleton model visualized using OpenSim^51^.

These subtle changes in coordination when using an active prosthesis may become clearer through an investigation of the contributions of each individual body segment to total *H* (Fig. 1), which is equal to the sum of each individual segment’s angular momentum relative to the body COM. Coordination of the body segments is critical for regulating balance, as the considerable arm and leg contributions to *H* largely cancel one another^18^, resulting in small overall values of *H*^19^. The additive property of *H* contrasts with other commonly used kinetic measures, such as net joint moment or power, which can be summed to provide an overall assessment of effort^20^ but are not truly independent quantities^21^.

Evaluating segment contributions to *H* can also help elucidate the complex relationship between ankle joint power generation and coordination of balance. Individuals with TTA have lost function in the soleus and gastrocnemius muscles, which have different kinetic effects on the leg and trunk^22^. Furthermore, pertaining to balance, the soleus and gastrocnemius generate sagittal-plane angular momentum in opposite directions during late stance. The soleus primarily creates a negative net external moment about the body center of mass, which acts to rotate the body forward (i.e., trunk moves forward and legs move backward), while the gastrocnemius creates a positive net external moment that rotates the body backward by accelerating the leg upward and forward for swing initiation^23^. In the frontal plane both the soleus and gastrocnemius contribute to angular momentum of the body toward the contralateral leg^24^. On ramps, the function of the gastrocnemius is largely unchanged, but the contributions of the soleus to the trunk vary^25^. Mechanically active ankle-foot prostheses can replicate the mechanical power generated at the ankle by the uniarticular soleus, but do not actuate the knee like the biarticular gastrocnemius. This structural difference may help explain why mechanically active prostheses have been found to give only modest improvements in regulating total *H* compared to a passive prosthesis on slopes despite increased ankle power generation^14^. In addition, with regard to balance it remains unclear which specific segments (i.e., legs, arms, trunk) are most affected by prosthetic ankle power generation and whether the changes in balance coordination are localized to the push-off phase of gait.

Most importantly, investigating segment contributions to *H* may also provide a practical tool for linking total *H* to specific mechanisms (e.g., coordination of individual body segments) that can be targeted in clinical gait retraining therapies. For example, it is straightforward to coach a patient to alter their joint mechanics (e.g., “Flex your knee more”), but it is difficult to understand quantitatively how these changes will affect coordination. Conversely, whole-body angular momentum is difficult to coach (e.g., “Reduce your sagittal-plane *H*”) but is directly related to balance. Altering momentum in a specific segment at a specific temporal location may be easier to explain (e.g., “Slow down your left arm during right leg stance”) and have a direct effect on coordination of balance. This type of targeted biofeedback could be made possible by an analysis that is capable of providing statistically valid assessments of coordination of balance.

To this end, our goal was to use Statistical Parametric Mapping (SPM) to analyze segment contributions to *H* in three dimensions in order to identify clinically relevant features of balance coordination during walking on various grades. We analyzed data from ten able-bodied individuals and ten individuals with unilateral transtibial amputation walking on ramp angles of 0°, ±5°,and ±10°. The individuals with amputation performed the protocol using a passive prosthesis as well as an active prosthesis (see Methods for further detail). We expected that use of the active prosthesis would result in upper body (trunk and arms) contributions to *H* that were significantly different compared to use of the passive prosthesis, but not significantly different from non-amputee participants during late stance and just after toe-off of the prosthetic leg. We expected this result because the active prosthesis generates mechanical power at the ankle joint in late stance, similar to the uniarticular soleus muscle that generates power to the trunk in late stance^22,25^. We also expected that the contributions of the prosthetic-side leg (using the active vs passive prosthesis) to *H* would be significantly different throughout gait due to the greater mass of the active prosthesis. Furthermore, we expected significant differences in the contributions from the prosthetic-side leg to *H* when using the active prosthesis compared to the non-amputee leg during late stance and toe-off. This expectation was based on the active prosthesis only actuating the ankle joint, and therefore being unable to replicate the function of the biarticular gastrocnemius in generating power to the leg to initiate swing^15,22^.

## Results

### Upper body: arms and trunk

**Figure 2:**
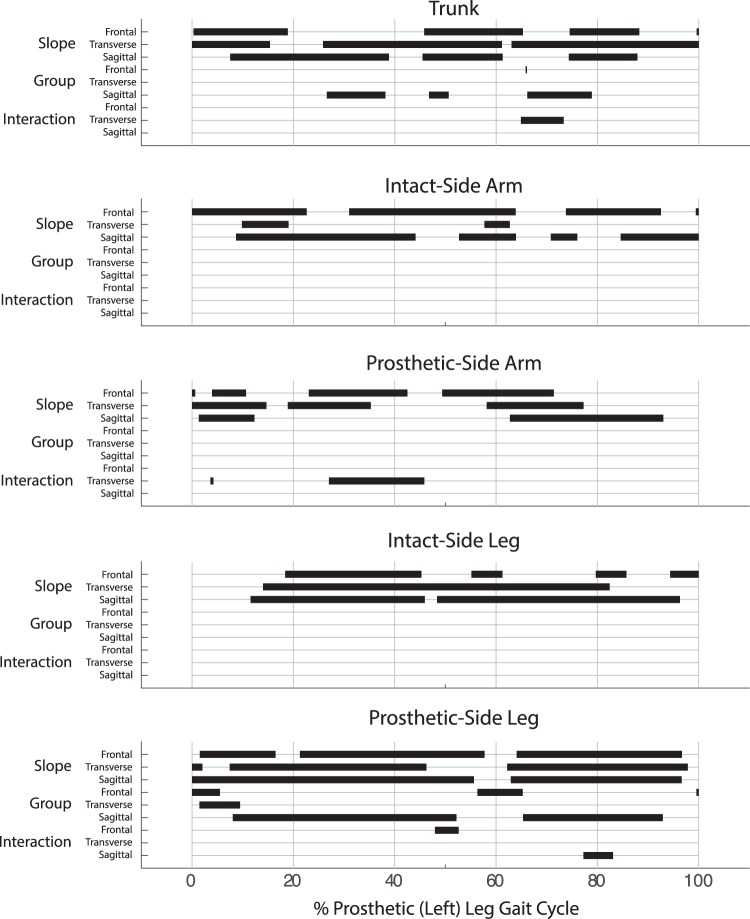
ANOVA results. Significant main (ramp angle, group) or interaction effects in each anatomical plane are indicated by solid bars spanning the region of the gait cycle where significant differences (α = 0.0033) were observed.

There was a significant main effect of ramp angle throughout large portions of the prosthetic/left leg gait cycle (gc) in all three anatomical planes for the arm and trunk contributions to *H* (Fig. 2). For the prosthetic-side arm, there were no group main effects. For the trunk, the group main effect was significant in the frontal plane just after prosthetic-side leg toe-off (65–66% gc, *p* = 0.003) and in the sagittal plane during mid to late prosthetic-side leg stance as well as just after prosthetic-side leg toe-off (26–38% gc, *p* < 0.001; 46–51% gc, *p* < 0.001; 66–79% gc, *p* < 0.001). There was a significant interaction effect for the prosthetic-side arm in the transverse plane during early (3–4% gc, p = 0.003) and mid (27–46% gc, *p* < 0.001) prosthetic-side leg stance, and in the trunk in the transverse plane just after prosthetic-side leg toe-off (65–73% gc, *p* < 0.001).

**Figure 3:**
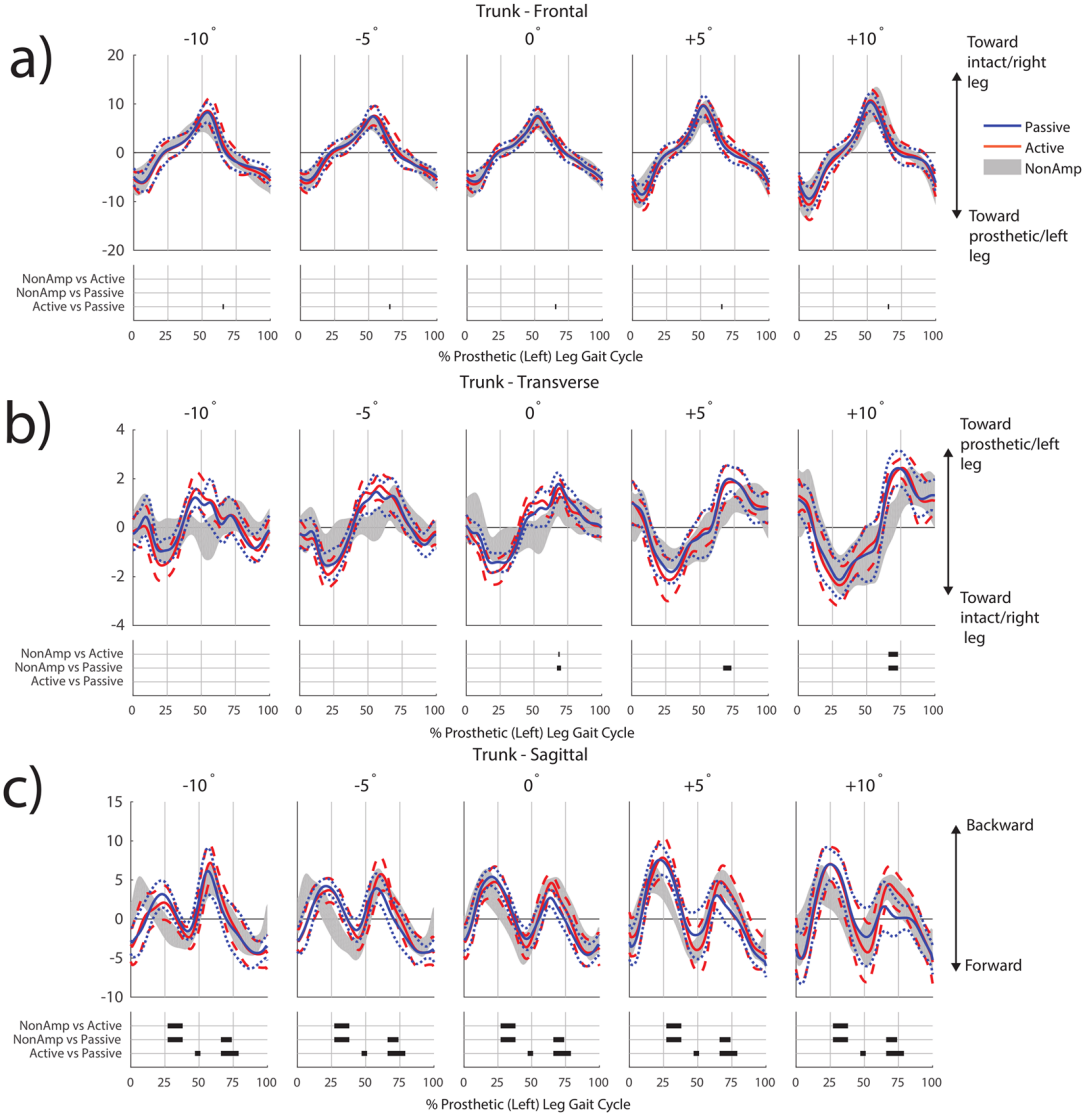
Contributions to normalized whole-body angular momentum from the trunk in the frontal (**a**), transverse (**b**) and sagittal (**c**) planes. Within each subfigure, the top row shows results for the passive prosthesis (mean in blue solid line, ±1 SD in blue dotted line) and active prosthesis (mean in orange solid line, ±1 SD in orange dashed line). Non-amputee data (mean ± 1 SD) are shown in shaded grey. Values have been normalized by participant height, mass, and average walking speed, and thus are unitless. Regions of statistical significance (α = 0.0005) from an SPM t-test are shown in solid bars on the bottom row of plots in each subfigure.

Post-hoc pairwise analyses of prosthetic-side arm contributions to *H* were not significant. Post-hoc pairwise analyses of trunk contributions to frontal-plane *H* showed a brief period of significantly increased contributions at toe-off (66% gc) on all grades for individuals with TTA using the active compared to passive prosthesis (Fig. 3a, Table 1). In the transverse plane, trunk contributions were more positive (toward prosthetic-side leg) just after toe-off in non-amputees compared to individuals with TTA using the active prosthesis at 0° and +10° ramp angles (Fig. 3b, Table 1). These differences just after toe-off were also present in non-amputees compared to individuals with TTA using a passive prosthesis at 0°, +5°, and +10°. In the sagittal plane, individuals with TTA had more positive trunk contributions to *H* (backward rotation) compared to non-amputees during mid stance when using both the active and passive prostheses on all slopes (Fig. 3c, Table 1). Just after toe-off, individuals with TTA using the passive prosthesis had more negative trunk contributions to *H* (forward rotation) compared to both non-amputees and the active prosthesis (Fig. 3c, Table 1).

**Table 1:** Pairwise comparison results for the trunk.

Trunk																
		−10			−5			0			5			10		
		region (%gc)	Peak SPM{t}	p-value	region (%gc)	Peak SPM{t}	p-value	region (%gc)	Peak SPM{t}	p-value	region (%gc)	Peak SPM{t}	p-value	region (%gc)	Peak SPM{t}	p-value
Frontal	NA vs A															
	NA vs P															
	A vs P	66.00–66.00	3.74	1.45E-07	66.00–66.00	3.74	1.45E-07	66.00–66.00	3.74	1.45E-07	66.00–66.00	3.74	1.45E-07	66.00–66.00	3.74	1.45E-07
Transverse	NA vs A							68.27–69.38*	−4.77	0.000453				66.09–73.00*	−4.71	0.0000765
	NA vs P							66.80–70.36*	−4.77	0.000235	67.21–73.00*	−4.71	0.000124	65.55–73.00*	−4.69	0.0000709
	A vs P															
Sagittal	NA vs A	27.00–38.00	−4.25	8.12E-12	27.00–38.00	−4.25	8.12E-12	27.00–38.00	−4.25	8.12E-12	27.00–38.00	−4.25	8.12E-12	27.00–38.00	−4.25	8.12E-12
	NA vs P	27.00–38.00 66.00–74.10	−4.24 4.24	2.22e-16 2.39e-05	27.00–38.00 66.00–74.10	−4.24 4.24	2.22e-16 2.39e-05	27.00–38.00 66.00–74.10	−4.24 4.24	2.22e-16 2.39e-05	27.00–38.00 66.00–74.10	−4.24 4.24	2.22e-16 2.39e-05	27.00–38.00 66.00–74.10	−4.24 4.24	2.22e-16 2.39e-05
	A vs P	47.00–51.00 66.00–79.00	−4.46 4.46	2.76e-06 3.77e-15	47.00–51.00 66.00–79.00	−4.46 4.46	2.76e-06 3.77e-15	47.00–51.00 66.00–79.00	−4.46 4.46	2.76e-06 3.77e-15	47.00–51.00 66.00–79.00	−4.46 4.46	2.76e-06 3.77e-15	47.00–51.00 66.00–79.00	−4.46 4.46	2.76e-06 3.77e-15

Comparisons are shown between non-amputees (NA), active prosthesis (A), and passive prosthesis (P). The region during which the SPM analysis indicated significant differences (α = 0.0005) are indicated as a percentage of gait cycle (gc). Regions in which significant differences were due to interaction effects are indicated with an asterisk (‘*’), all other regions were main effects. The peak SPM{t} for each region is also reported. Lastly, p-values for each region are reported. Empty cells indicate no significant differences.

### Lower body: legs

There was a significant main effect of ramp angle in both the intact- and prosthetic-side leg during large portions of the gait cycle (Fig. 2). In the intact-side leg, there were no significant group main effects or interaction effects. In the prosthetic-side leg, there were significant group main effects in the frontal plane during early stance (0–6% gc, *p* < 0.001;), around toe-off (56–65% gc, *p* < 0.001), and just before heel strike (99–100% gc, *p* = 0.003). In the transverse plane, there were significant group main effects during early stance (1–9% gc, *p* < 0.001). In the sagittal plane, there were significant group main effects during large portions of stance (8–52% gc, *p* < 0.001) and swing (65–93% gc, *p* < 0.001). There were significant interaction effects in the frontal plane during late stance (48–53% gc, *p* < 0.001) and in the sagittal plane during swing (77–83% gc, *p* < 0.001).

**Figure 4:**
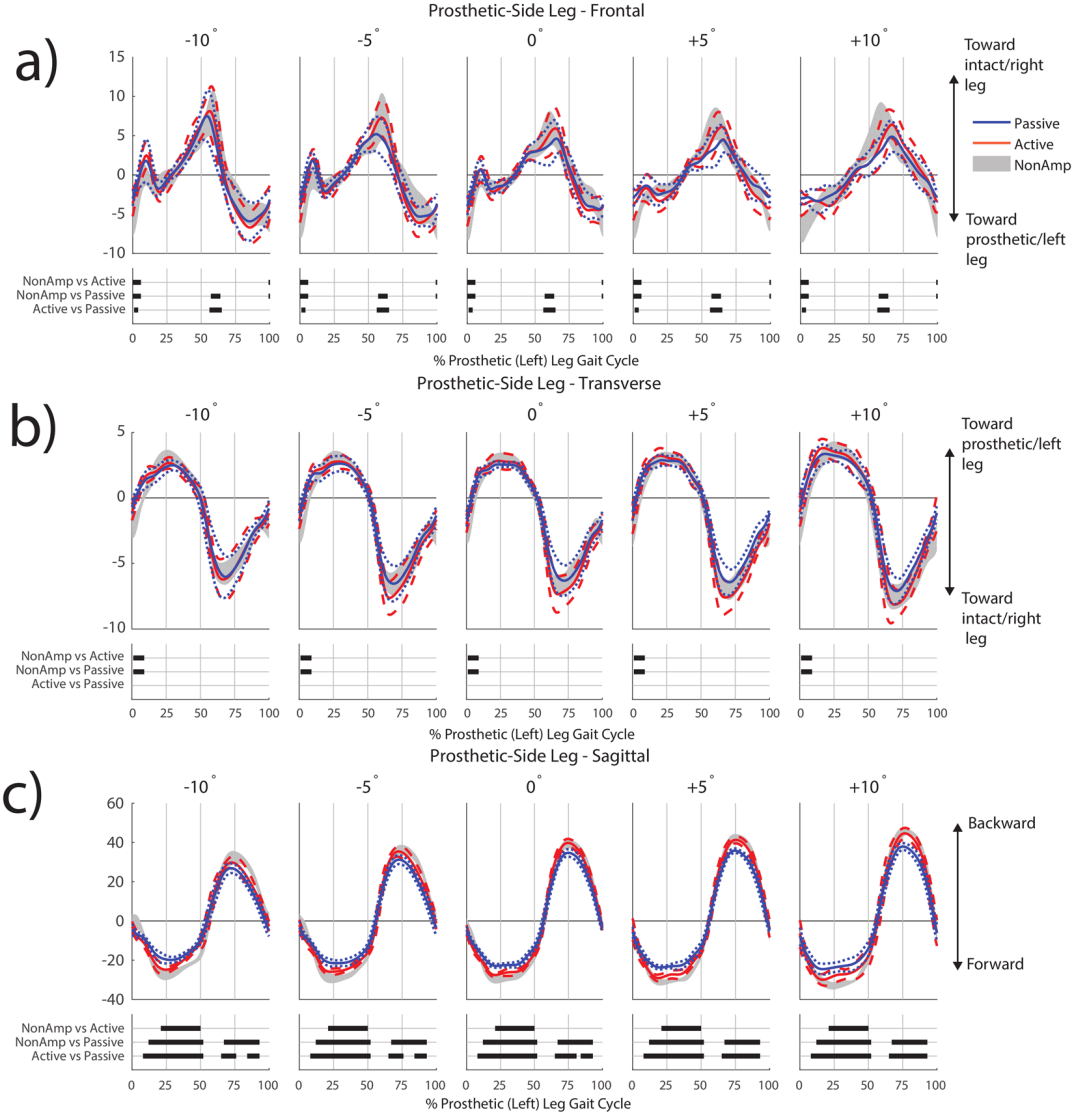
Contributions to normalized whole-body angular momentum from the prosthetic-side (left) leg in the frontal (**a**), transverse (**b**) and sagittal (**c**) planes. Within each subfigure, the top row shows results for the passive prosthesis (mean in blue solid line, ±1 SD in blue dotted line) and active prosthesis (mean in orange solid line, ±1 SD in orange dashed line). Non-amputee data (mean ± 1 SD) are shown in shaded grey. Values have been normalized by participant height, mass, and average walking speed, and thus are unitless. Regions of statistical significance (α = 0.0005) from an SPM t-test are shown in solid bars on the bottom row of plots in each subfigure.

Post-hoc pairwise comparisons indicated that the prosthetic-side leg contributions to frontal-plane *H* were more positive (toward intact leg) in individuals with TTA using both the active and passive prostheses compared to non-amputees in early stance and just before heel strike (Fig. 4a, Table 2). In addition, the active prosthesis resulted in prosthetic-side leg contributions that were more positive than the passive prosthesis during early stance. At toe-off, the passive prosthesis resulted in prosthetic-side leg contributions that were significantly lower (less rotation toward intact leg) compared to both non-amputees and the active prosthesis (Fig. 4a, Table 2).

**Table 2:** Pairwise comparison results for the prosthetic-side (left) leg.

Prosthetic Side Leg																
		−10			−5			0			5			10		
		region (%gc)	Peak SPM{t}	p-value	region (%gc)	Peak SPM{t}	p-value	region (%gc)	Peak SPM{t}	p-value	region (%gc)	Peak SPM{t}	p-value	region (%gc)	Peak SPM{t}	p-value
Frontal	NA vs A	0.00–6.00 100.00–100.00	−4.14 −4.14	2.80e-06 2.93e-10	0.00–6.00 100.00–100.00	−4.14 −4.14	2.80e-06 2.93e-10	0.00–6.00 100.00–100.00	−4.14 −4.14	2.80e-06 2.93e-10	0.00-6.00 100.00-100.00	−4.14 −4.14	2.80e-06 2.93e-10	0.00–6.00 100.00–100.00	−4.14 −4.14	2.80e-06 2.93e-10
	NA vs P	0.00–6.00 57.34–63.65 100.00–100.00	−4.14 4.14 −4.14	7.78e-09 7.51e-05 2.44e-15	0.00-6.00 57.34–63.65 100.00–100.00	−4.14 4.14 −4.14	7.78e-09 7.51e-05 2.44e-15	0.00–6.00 57.34–63.65 100.00–100.00	−4.14 4.14 −4.14	7.78e-09 7.51e-05 2.44e-15	0.00-6.00 57.34-63.65 100.00–100.00	−4.14 4.14 −4.14	7.78e-09 7.51e-05 2.44e-15	0.00–6.00 57.34–63.65 100.00–100.00	−4.14 4.14 −4.14	7.78e-09 7.51e-05 2.44e-15
	A vs P	0.76–3.73 56.00–65.00	−4.38 4.38	2.45e-04 2.80e-08	0.76–3.73 56.00–65.00	−4.38 4.38	2.45e-04 2.80e-08	0.76–3.73 56.00–65.00	−4.38 4.38	2.45e-04 2.80e-08	0.76–3.73 56.00–65.00	−4.38 4.38	2.45e-04 2.80e-08	0.76–3.73 56.00–65.00	−4.38 4.38	2.45e-04 2.80e-08
Transverse	NA vs A	1.00–9.00	−3.87	4E-13	1.00–9.00	−3.87	4E-13	1.00–9.00	−3.87	4E-13	1.00–9.00	−3.87	4E-13	1.00–9.00	−3.87	4E-13
	NA vs P	1.00–9.00	−3.87	2E-16	1.00–9.00	−3.87	2E-16	1.00–9.00	−3.87	2E-16	1.00–9.00	−3.87	2E-16	1.00–9.00	−3.87	2E-16
	A vs P															
Sagittal	NA vs A	20.85–49.68	−4.39	0	20.85–49.68	−4.39	0	20.85–49.68	−4.39	0	20.85–49.68	−4.39	0	20.85–49.68	−4.39	0
	NA vs P	11.79–52.00 67.12–93.00 77.00–83.00*	−4.38 4.38 4.70	0.00e + 00 1.50e-13 3.67e-06	11.79–52.00 67.12–93.00 77.00–83.00*	−4.38 4.38 4.68	0.00e + 00 1.50e-13 3.65e-06	11.79–52.00 67.12–93.00 77.00–83.00*	−4.38 4.38 4.68	0.00e + 00 1.50e-13 3.04e-05	11.79–52.00 67.12–93.00 77.00–83.00*	−4.38 4.38 4.68	0.00e + 00 1.50e-13 2.44e-11	11.79–52.00 67.12–93.00 77.00–83.00*	−4.38 4.38 4.63	0.00e + 00 1.50e-13 8.72e-11
	A vs P	8.00–52.00 65.00–93.00	−4.69 4.69	0.00e + 00 0.00e + 00	8.00–52.00 65.00–93.00	−4.69 4.69	0.00e + 00 0.00e + 00	8.00–52.00 65.00–93.00 77.00–80.68*	−4.69 4.69 6.25	0.00e + 00 0.00e + 00 1.06e-04	8.00–52.00 65.00–93.00 77.00–83.00*	−4.69 4.69 6.29	0.00e + 00 0.00e + 00 1.72e-09	8.00–52.00 65.00–93.00 77.00–83.00*	−4.69 4.69 6.27	0.00e + 00 0.00e + 00 4.25e-06

Comparisons are shown between non-amputees (NA), active prosthesis (A), and passive prosthesis (P). The region during which the SPM analysis indicated significant differences (α = 0.0005) are indicated as a percentage of gait cycle (gc). Regions in which significant differences were due to interaction effects are indicated with an asterisk (‘*’), all other regions were main effects. The peak SPM{t} for each region is also reported. Lastly, p-values for each region are reported.

In the transverse plane, post-hoc pairwise comparisons indicated that prosthetic-side leg contributions were more positive (toward prosthetic-side leg) with both the active and passive prostheses compared to non-amputees during early stance on all ramp angles (Fig. 4b, Table 2). There were no differences between the passive and active prostheses.

In the sagittal plane, post-hoc pairwise comparisons indicated differences between all groups during large portions of stance (Fig. 4c, Table 2). The passive prosthesis resulted in the most positive (backward) contributions to *H*. The active prosthesis provided more negative (forward) contributions, but still not as much as non-amputees. The region of significant difference appeared to be smaller in individuals with TTA using the active prosthesis compared to non-amputees (21–50% gc) than in non-amputees compared to individuals with TTA using the passive prosthesis (12–52% gc). In addition, the passive prosthesis had a smaller contribution to positive (backward) *H* during swing (~67–93% gc) compared to both non-amputees and the active prosthesis (Fig. 4c, Table 2).

## Discussion

This study analyzed data from one of the first major clinical studies of a mechanically active ankle-foot prosthesis on ramps. Our goal was to use SPM, a novel statistical method for analyzing biomechanical data, to extend prior analyses of the range of *H*^14^ to identify aspects of time-varying three-dimensional segmental coordination of balance (i.e., *H*) that are clinically relevant. We expected use of the active prosthesis to result in upper body (trunk and arms) contributions to *H* that were not significantly different in comparison to non-amputee participants during late stance and just after toe-off of the prosthetic leg. We also expected the contributions of the prosthetic leg to *H* would be significantly different between the passive and active prostheses, as well as between the active prosthesis and the average non-amputee leg.

Our first expectation was partially supported, as the active prosthesis altered coordination of the trunk but not the intact- or prosthetic-side arm. In the frontal plane, there was a brief period when the trunk contributions to *H* were more positive when using the active compared to passive prosthesis, indicating greater angular momentum toward the intact leg side at the time of prosthetic leg toe-off (66% gc). The timing of these changes in trunk coordination corresponded with greater positive frontal plane *H* from the prosthetic-side leg (56–65% gc) with the active compared to passive prosthesis, suggesting that the active generation of ankle joint power contributes additional frontal-plane angular momentum of the prosthetic leg and trunk toward the intact leg. These findings are supported by a number of other studies demonstrating increased ankle push-off power^2,3^ and trailing leg mechanical work^5,6^ when using an active compared to a passive prosthesis. Our findings also provide experimental evidence to support musculoskeletal simulations demonstrating that the ankle plantarflexors contribute to frontal-plane *H* toward the contralateral leg in late stance^24^. Our analysis expands upon prior results by identifying that the ipsilateral leg and trunk are the primary segments affected by active ankle power generation on various ramp angles.

Our findings in the sagittal plane were similar to those in the frontal plane. There were no differences in the trunk at toe-off between non-amputees and people with TTA using an active prosthesis, whereas there were significant differences between people with TTA using a passive prosthesis compared to either the active prosthesis or non-amputees. Particularly with respect to the trunk, this finding is consistent with the role of the uniarticular soleus muscle, which generates mechanical power to the trunk segment during late stance on level ground^22^ and inclines^25^. The increased positive angular momentum of the trunk at the time of prosthetic leg toe-off can be thought of as backward angular momentum, with the upper body moving posteriorly while the lower body moves anteriorly relative to the body COM. Although we did not evaluate muscle activity in this study, it is possible that this backward angular momentum may reduce the demand on the hip extensors of people with transtibial amputation, particularly when walking uphill. The hip extensors help initiate contralateral leg swing by transferring mechanical power from the trunk to both legs on inclines^25^. These results are also supported by musculoskeletal simulation results indicating that use of a mechanically active compared to passive prosthesis reduces the amount of power generated to the trunk and both legs by the hamstrings in the amputated leg^15^. All of these results taken together suggest benefits in trunk coordination during ramp walking due to active ankle power generation.

However, despite changes that indicate improved (i.e., more similar to non-amputees) segmental coordination of dynamic balance in the sagittal and frontal planes during toe-off when using an active compared to a passive prosthesis, other potentially detrimental gait characteristics persisted regardless of whether the prosthesis was active or passive. Both passive and active prostheses resulted in increased positive (backward) trunk contributions to sagittal-plane *H* during mid stance (27–38% gc) relative to non-amputees. During this portion of the gait cycle the active prosthesis does not generate ankle power, so the two types of prosthetic feet both function passively. This increased backward angular momentum likely contributes to the relatively small differences between prostheses in the range of total sagittal-plane *H* during 0–50% gc that have been previously observed^14^. Our results are also consistent with prior studies of uphill walking, which found that active and passive prostheses behaved similarly during loading of the prosthetic leg^2^. One possible explanation for this is that maintaining balance or stability can conflict with other task objectives during walking. People with TTA may compromise stability during early- and mid-stance in favor of energetic objectives such as minimizing metabolic cost^26^. Alternatively, factors such as muscle atrophy in the amputated limb^27^ or reduced proprioception in the prosthesis compared to a biological leg may impair the ability to regulate *H*. The interface between the socket of the prosthesis and the amputated leg may also be impair segmental coordination of balance with an active prosthesis^28^, potentially due to pressure or pain in the socket^29^. Specific gait retraining may be necessary to help people with TTA alter their gait patterns during early- and mid-stance and better take advantage of active ankle power generation, potentially using human-in-the-loop optimization of device parameters^30^.

Both passive and active prostheses resulted in more positive (toward the intact leg) trunk contributions to transverse-plane *H* at toe-off (~68–70% gc) when compared to non-amputees on level ground and the +10° ramp. The greater transverse *H* toward the amputated leg during stance is similar to previous results from Gaffney *et al*., who analyzed whole-body angular momentum relative to the foot^31^. Gaffney *et al*. attributed this increase in *H* to a wider stance, thereby increasing the contributions of the trunk and pelvis velocity relative to the stance foot. The present results extend this finding by showing that transverse-plane *H* is greater when computed relative to the body COM as well. This greater transverse-plane *H* may be related to alterations in torso-pelvis coordination, as people with transtibial amputation have excessive trunk rotation toward the intact leg during walking^32^ and toward the amputated leg during sit-to-stand^33^. Altered trunk coordination may be a contributing factor to chronic low back pain in people with transtibial amputation^34^. While this relationship warrants further investigation, our results highlight the fact that clinically relevant changes in segmental coordination that are overlooked by an analysis of range of *H* can be identified as statistically significant using SPM. Identifying the temporal features of these changes in segmental coordination is important because transverse-plane *H* is small in magnitude, and conventional analyses of range of *H* often fail to identify statistically significant results in this plane^14^.

A key advantage of an SPM analysis over scalar analyses of values such as minimum, maximum, or range, is that it enables development of targeted interventions. For example, a clinical professional may direct a patient to reduce the rotation of their trunk during prosthetic-side leg toe-off. This instruction is targeted to a specific part of the body at a specific time in the gait cycle. Future work should be directed at determining whether this type of targeted balance rehabilitation can reduce secondary conditions, such as low back pain, over time. Assistive devices that specifically target segmental coordination of balance, or operate based on principles of balance (such as robotic gyrosuit devices that can provide angular momentum assistance at the trunk^35^) rather than only kinetics or kinematics at the ankle joint, may aid in these types of rehabilitation programs.

In support of our expectation regarding leg contributions to *H*, there were many differences in prosthetic-side/left leg contributions to *H* between all three participant groups in all three anatomical planes during various portions of the gait cycle (Fig. 4). Both the passive and active prostheses were significantly different from non-amputees early in the gait cycle, further demonstrating that generating ankle joint power in late stance is not sufficient to fully restore able-bodied gait patterns.

A factor that likely influences the differences between passive and active prostheses is mass. Passive prostheses are lighter than active prostheses, and in our dynamic model we accounted for this difference by reducing the shank mass and moving the shank COM proximally (toward the knee joint) in the passive prosthesis model. If the differences we observed in prosthetic leg contributions to *H* were entirely due to mass differences, the greater mass of the powered prosthesis could have detrimental effects such as increased demand on the hip flexors and extensors. In addition, the greater mass of an active prosthesis may increase metabolic cost due to the distal placement of the mass^36^. However, the changes in trunk and prosthetic-side leg contributions to *H* near toe-off suggest that active ankle power generation, and not only inertial differences, play a key role in the observed changes in *H*. Recently developed prosthesis emulators^37^ may be able to decouple the effects of ankle power generation and increased mass, which would be a useful area of future study.

There were no significant group or interaction effects in the intact-side arm or leg (Fig. 2). Gait asymmetry in quantities related to balance (e.g., margin of stability^38^) and compensatory kinematic strategies to overcome prosthesis limitations (e.g., increased prosthetic-side hip flexion^39^), are frequently noted as detrimental outcomes of TTA. A change in angular momentum of the prosthetic-side arm or leg may be expected to result in an opposite change on the intact side of the body, but our results did not show this contralateral effect. One possible explanation is that prosthesis users are unable to effectively transfer mechanical energy generated by active prostheses to the contralateral side of the body. This may be due to a number of factors, including energy dissipation or discomfort at the socket interface^29^ or altered hamstrings function in people with TTA^15^. Future studies should investigate whether specific gait retraining can increase the amount of energy transmitted to the contralateral side and reduce the asymmetry in the upper body.

Potential limitations of this study include assumptions in inertial properties of the body segments, which may affect the calculations of segment contributions to *H*, particularly in the prosthesis and amputated leg. We accounted for the reduced mass of the passive prosthesis in accordance with published experimental data regarding prosthesis inertial properties^40^. However, the size and shape of the residual leg varies between individuals, and its inertial properties are difficult to determine. Given that we identified a number of differences in contributions of the prosthetic-side leg to *H* between groups, the effects of prosthetic-side leg inertial properties on coordination of balance warrants further investigation. In addition, it is important to note that our sample is not truly random in that we previously analyzed the range of *H* in the same participant group. We used conservative corrections (e.g., Bonferroni) to increase confidence in our results, but future prospective studies would be useful to further corroborate our findings.

In conclusion, SPM allows for detailed analysis of 3D coordination of balance during walking on ramps. Specifically, our SPM analysis revealed that the addition of active prosthetic ankle power generation largely restores trunk and prosthetic-side leg coordination to non-amputee patterns around toe-off. These changes are consistent with the functional role of the uniarticular soleus, which contributes to trunk acceleration during walking. However, the SPM analysis also revealed that differences relative to non-amputees persist at other points in the gait cycle even when using a mechanically active prosthesis that provide physiologically appropriate levels of ankle power. The cause of these persistent balance differences remains unclear, and future research should investigate the possible roles of the socket-limb interface, intrinsic compensatory movement patterns, and muscle weakness in contributing to these persistent differences. The method used in this paper are complementary to assessments of metabolic cost and joint kinetics, and can identify temporal features of segmental coordination of balance that are clinically and statistically significant. These results are valuable for informing future studies of real-time biofeedback of *H* to enable targeted clinical retraining of specific segments to improve coordination of dynamic balance.

## Methods

### Experimental Data Collection

We evaluated data from ten individuals with TTA (1 female/9 male, mean ± SD age 30 ± 5 years, height 1.83 ± 0.10 m, body mass 96 ± 7 kg) and ten non-amputees (2 female/8 male, mean ± SD age 24 ± 5 years, height 1.80 ± 0.09 m, body mass 91 ± 10 kg). The data collected in this study represent the first large-scale third-party evaluation of a commercially available active ankle-foot prostheses. The kinematics and kinetics^2^ and range of *H*^14^ have been previously reported for these data, but timeseries segmental contributions to *H* have not yet been reported. All participants provided written informed consent to participate in the experimental protocol, which was approved by the Institutional Review Board at Brooke Army Medical Center, and the experimental procedures were carried out in accordance with the relevant regulations and guidelines. Inclusion criteria for individuals with transtibial amputation were Medicare Functional Classification Level K4^41^, ability to walk independently for 15 consecutive minutes, history of independent ambulation on slopes for at least one month prior to the study (mean ± SD 18.4 ± 11.1 months), Visual Analog Scale pain scores on the involved side of less than 4/10, and normal range of motion in all unaffected joints. The exclusion criteria for individuals with TTA included blindness, traumatic brain injury, unhealed wounds, active infection, pregnancy, and cardiac or pulmonary conditions limiting physical activity. Non-amputee participants were selected to be age-, weight-, and height-matched to participants with transtibial amputation. Non-amputee participants were excluded on the basis of a known history of balance or visual impairment, neurological disorders, chronic musculoskeletal conditions, cardiac or pulmonary conditions.

Whole-body kinematics were recorded at a frequency of 120 Hz (Motion Analysis Corp., Santa Rosa, CA) while participants walked on an adjustable 16-foot ramp at grades of 0°, ±5°, and ±10°. The grades were presented in randomized order. Participants with transtibial amputation walked first with their clinically prescribed passive prosthesis, then with a pre-release version of the BiOM H2 Power Ankle (BiOM, Inc., Bedford, MA) active prosthesis on a later visit. A prosthetist adjusted the magnitude and timing of mechanical power delivered by the active prosthesis to match normative ankle mechanics (within ±2SD) on level ground, described in detail by Rabago *et al*.^2^. Subjective walking assessments were given during a practice session with the active prosthesis, and participants were instructed to practice uphill and downhill walking during a mean acclimation period of 43.4 (SD = 18.1) days. We controlled horizontal velocity (Froude number 0.16) using an auditory cue^42^. The resulting mean horizontal velocity was 1.28 (SD = 0.11) m/s for individuals with TTA and 1.21 (SD = 0.08) m/s for non-amputees. 57 reflective markers were used to define and track 13 body segments^43,44^. Kinematic marker trajectories were filtered using a 4th-order low pass Butterworth filter with a 6 Hz cutoff frequency.

Simplified geometric models of each person were developed in Visual3D (C-Motion, Inc., Germantown, MD), with segment masses computed based on total body mass^45^. The mass of the shank was reduced by 30% and moved 30% more proximal to model the inertial properties of the passive prostheses^40^. The active prosthesis was assumed to have the same inertial properties as a biological shank and foot due their similar mass^46^. The contribution of each segment to *H* was calculated as

2 $${\mathop{H}\limits^{\rightharpoonup }}_{i}=({\mathop{r}\limits^{\rightharpoonup }}_{i}-{\mathop{r}\limits^{\rightharpoonup }}_{body})\times {m}_{i}({\mathop{v}\limits^{\rightharpoonup }}_{i}-{\mathop{v}\limits^{\rightharpoonup }}_{body})+{I}_{i}{\mathop{\omega }\limits^{\rightharpoonup }}_{i}$$

where ${\mathop{r}\limits^{\rightharpoonup }}_{i}$, ${\mathop{v}\limits^{\rightharpoonup }}_{i}$ and ${\mathop{\omega }\limits^{\rightharpoonup }}_{i}$ are, respectively, the position, velocity, and angular velocity of the ${i}^{th}$ segment, ${\mathop{r}\limits^{\rightharpoonup }}_{body}$ and ${\mathop{v}\limits^{\rightharpoonup }}_{body}$ are, respectively, the position and velocity of the whole-body COM, and ${m}_{i}$ and ${I}_{i}$ are the mass and inertia matrix of the ${i}^{th}$ segment. Each segment contribution *H*_*i*_ was normalized by body height, mass, and average horizontal walking velocity and expressed as a percentage of the left or prosthetic limb gait cycle for the non-amputee and TTA groups, respectively.

### Statistical Analysis

To detect clinically relevant features of segmental coordination at each point in the gait cycle, the contributions of each arm (forearm, upper arm), leg (thigh, shank, foot) and the trunk (head, torso, pelvis) to *H* throughout the gait cycle were compared using Statistical Parametric Mapping (SPM). SPM enables statistical comparisons between entire trajectories rather than selected features from those trajectories^47^. Originally developed for analyzing functional brain images^48^, SPM is performed by computing a test statistic (e.g., Student’s *t*, Fisher’s *F*) at each time point. Random field theory is then used to determine the threshold for significance while maintaining a family-wise error rate of α^47^. Thus, rather than analyzing single values, such as range, the entire trajectory of biomechanical data can be analyzed while properly controlling for multiple comparisons and dependence between neighboring time points. In our analysis, we implemented custom MATLAB code based on the spm1d package^49^. A generalized linear model was created with ramp grade and group (non-amputee, passive, active) as fixed effects. We also included a random intercept effect for each participant, as well as random slope effects for each main interaction effect. The random effects were assumed to be uncorrelated. For each participant, data from four complete gait cycles were collected at each ramp angle, which were then averaged together prior to analysis.

The model was applied to the contributions to *H* in the three anatomical directions for each segment separately. An ANOVA was performed to test the significance of the model coefficients at each time step. The time trajectory of the resulting *F*-statistics throughout the gait cycle is referred to as SPM{*F*}. The threshold for significance was determined based on the full-width half-maximum of the residuals in the linear model, as well as a corrected α=0.0033 due to a total of 15 ANOVAs being performed. The portions of the SPM{*F*}, which lie above the critical threshold are referred to as suprathreshold clusters. A *p*-value was computed for each cluster to represent the probability that smooth, Gaussian random fields of equivalent full-width half-maximum would produce a test statistic field that surpassed the threshold to yield a cluster at least as large as the observed one. The denominator degrees of freedom for the fixed effects were estimated at each time point using the Satterthwaite approximation, and the average value across all time steps was used in the SPM analysis.

When significant main effects were found, *post hoc* pairwise comparisons were performed. A paired *t*-test was used to compare between passive and active prosthesis trials, and an unpaired *t*-test was used to compare non-amputees and individuals with TTA. Similar to the ANOVA results, the analysis produced a time trajectory of SPM{*t*} values. If only the main effect was significant in the ANOVA for a given time point, we report the pooled comparison results. If the ANOVA interaction term was significant, the results from a full comparison of each group/ramp grade combination are presented. Pairwise comparisons were performed only during regions of interest^50^ in the gait cycle in which significant main or interaction effects were found in the ANOVA. We used a conservative Bonferroni correction to adjust the significance level based on a full analysis of all 3 groups × 5 slopes = 15 means, or 105 total comparisons, giving a critical threshold of *p*_*critical*_ = 0.0005.

## Data Availability

The datasets generated during and/or analyzed during the current study are not publicly available due to confidentiality regulations pertaining to data collected at Brooke Army Medical Center, but may be available if approved by the appropriate entity.
